# Supplementary material for: Fingerprints of supersymmetric spin and charge dynamics observed by inelastic neutron scattering
Source: Nat Commun. 2025 Apr 4;16:3228. doi: 10.1038/s41467-025-58380-7 (PMC11971275; doi:10.1038/s41467-025-58380-7)
Supplement: Supplementary file 1 — Supplementary Information [file 41467_2025_58380_MOESM1_ESM.pdf]

Supplementary Information

to accompany the article

Fingerprints of supersymmetric spin and charge dynamics observed by inelastic neutron scattering

Björn Wehinger, Franco T. Lisandrini, Noam Kestin, Pierre Bouillot, Simon Ward, Benedikt Thielemann, Robert Bewley, Martin Boehm, Daniel Biner, Karl W. Krämer, Bruce Normand, Thierry Giamarchi, Corinna Kollath, Andreas M. Läuchli and Christian Rüegg

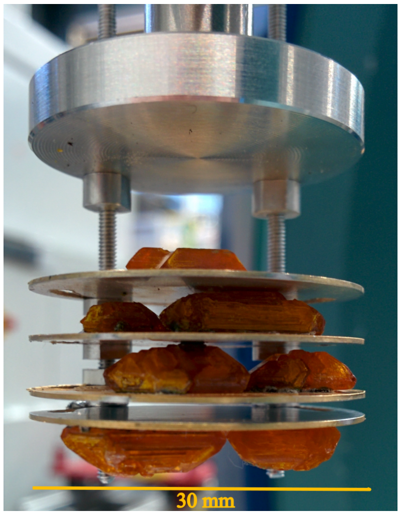

Supplementary Figure 1. **BPCC sample.** Photograph of the mounted BPCC crystal ensemble used in the INS experiment on LET.

Supplementary Note 1. SPIN-LADDER MATERIALS

High-quality deuterated single crystals of both bis-piperidinium copper (II) bromide (BPCB) and chloride (BPCC) were grown by slow solvent evaporation at a fixed temperature of 25 °C following the method established previously.[1] For BPCB, an ethanol solution (C_2H_5OD with D > 99%) with deuterated piperdinium bromide (D > 99%) and copper bromide was used. Large crystals with improved quality were obtained by mixing two moles of copper bromide and one mole of fully deuterated piperdinium bromide in ethanol solution with a few drops of hydrobromic acid, DBr (D > 99%) to avoid hydrolysis. The solution was evaporated slowly in a flow of dry nitrogen gas and suitable crystals were obtained after 2-3 months. BPCC crystals were grown following the same procedure by replacing the bromides with the respective chloride compounds.

For both experiments, several crystals were co-aligned within 1° with the aid of neutron diffraction on the MORPHEUS instrument at the Swiss Spallation Neutron Source, SINQ, and were glued onto a gold-coated aluminum holder to prevent any chemical reactions. The mount for the BPCB experiment consisted of six co-aligned crystals with a total mass of 1.4 g. Supplementary Fig. 1 shows the mount of seven crystals with a total mass of 3.535 g used in the BPCC experiment.

BPCB and BPCC are isostructural, crystallizing in the monoclinic space group P2$_1$/c.The structure of BPCB was determined by neutron diffraction at 1.8 K[2] to have lattice parameters a = 8.371 Å, b = 16.939 Å and c = 12.382 Å, with monoclinic angle $\beta = 101.20°$. For BPCC the lattice parameters were determined at 1.6 K[3] to be a = 8.0866 Å, b = 16.577 Å and c = 12.145 Å, with monoclinic angle $\beta = 102.24°$. The $\mathbf{a}$ axis is the ladder direction for both materials and the rung alignment vectors are $\mathbf{r}_\pm = (0.3910, \pm 0.1625, 0.4810)$ for BPCB and $\mathbf{r}_\pm = (0.3822, \pm 0.1730, 0.4866)$ for BPCC, expressed in fractions of the unit cell.

Supplementary Note 2. INELASTIC NEUTRON SCATTERING

Inelastic Neutron Scattering (INS) measures the combination of dynamical structure factors

$$\frac{d^2\sigma}{d\Omega dE'} \propto \frac{q'}{q}|F(\mathbf{Q})|^2\Big\{\Big(1-\frac{Q_z^2}{\mathbf{Q}^2}\Big)\Big[c(\mathbf{Q})S_0^{zz}+s(\mathbf{Q})S_\pi^{zz}\Big] + \Big(1+\frac{Q_z^2}{\mathbf{Q}^2}\Big)\Big[c(\mathbf{Q})S_0^{xx}+s(\mathbf{Q})S_\pi^{xx}\Big]\Big\}, \quad (1)$$

in which $\mathbf{Q}$ is the momentum transfer, ω the energy transfer, $c(\mathbf{Q}) = \sum_{m=1}^2 \cos^2\left(\frac{1}{2}\mathbf{Q}\cdot\mathbf{r}_m\right)$, $s(\mathbf{Q}) = \sum_{m=1}^2 \sin^2\left(\frac{1}{2}\mathbf{Q}\cdot\mathbf{r}_m\right)$, $m = 1,2 \equiv \pm$ denotes the two inequivalent ladder rung vectors $\mathbf{r}_\pm$ (Supplementary Note 1) and $F(\mathbf{Q})$ is the form factor of the Cu^{2+} ion. $S_{q_\perp}^{\alpha\gamma}$ is the dynamical structure factor for sector $q_\perp$, which is defined by

$$S_{q_\perp}^{\alpha\gamma}(q_\parallel,\omega) = \sum_\ell \int dt\, e^{+i(\omega t - q_\parallel x_\ell)}\big\langle S_{\ell,q_\perp}^\alpha(t) S_{0,q_\perp}^\gamma(0)\big\rangle, \quad (2)$$

where $\alpha,\gamma \in \{x,y,z,+,-\}$ and $S_{\ell,q_\perp}^\alpha \equiv S_{\ell,1}^\alpha \pm S_{\ell,2}^\alpha$ are the symmetric (+) and antisymmetric (−) operators with respective rung momenta $q_\perp = 0$ and π. The correlation functions in Supplementary Eq. (2) are evaluated at $q_\parallel = \mathbf{Q}\cdot\mathbf{a}$. Supplementary Table 1 shows the triplet modes contributing to the scattering cross-sections in each spin and parity sector.

A. BPCB measurements on IN14

The BPCB experiment was performed at the triple-axis spectrometer ThALES at the Institut Laue-Langevin (ILL), which is equipped with a 15 T vertical-field magnet and a dilution refrigerator insert. The final neutron momentum of 1.3 $\mathrm{\AA}^{-1}$ was set by a PG(002) monochromator with a Be filter, which provided focusing in both the horizontal and vertical directions. The sample was orientated with its c axis parallel to the magnetic field and the measured range in $q_{\|} \equiv 2\pi q_h$ was optimized for the resolution ellipsoid, which was estimated from measurements of the triplet excitations at $B^z = 0$ and 6 T at nine distinct q_h values between 0.5 and 1.0. Values for the FWHM in both q_h and E were obtained from Gaussian fits. The momentum- and energy-dependence of these widths were found to be small and thus the resolution function used for convolution with the data from our numerical calculations was assumed to be constant in q_h and E, inclined by a fixed positive angle. The temperature was set to 50 mK and the scattering intensities collected at the chosen q_h and field values were normalized to the monitor value of the incoming flux. By measuring the signal in regions of E and q_h far from any magnetic excitations, the background arising from detector noise was found to be well approximated by a constant.

B. BPCC measurements on LET

The BPCC experiment was conducted on the LET spectrometer[4] at ISIS (Rutherford Appleton Laboratory). This instrument has a customized 9 T vertical magnet with wide-angle Al windows for incoming and scattered neutrons. The incoming neutron energy was 2.5 meV, the vertical field was set parallel to the sample c axis, with $B^z = 2.876$ T ensuring half-polarization, and the temperature was controlled by a dilution refrigerator. The sample was rotated around the c axis in 1° steps and data were collected until a monitor count of 30 μAs at each of 104 angular positions. The energy resolution of LET varies with the scattering angle, and for the parameters of the BPCC experiment changed with q_h and E as quoted in the Methods section, namely from 37 μeV at $E = 0$ to 28 μeV at $E = 0.9$ meV for $q_h = 0$ and from 56 μeV to 34 μeV at $q_h = -1$. The high momentum resolution (0.05 r.l.u. along a^*) available at LET, which allowed a full comparison of the two $q_\perp$ sectors, was a primary reason for performing the experiment on this spectrometer. Scattering intensities were corrected for detector efficiency and scattered-to-incident wavevector ratio using the MANTID code[5] and were binned into $S(\mathbf{Q},\omega)$ datasets. We processed these data by constructing reciprocal-space cuts to select the independent excitation channels using a mask for the different $q_\perp$ sectors.[3]

All figures shown in the main text have full background subtraction. For completeness we show in Supplementary Fig. 2 the raw data from LET from which our BPCC spectra were obtained. We remind the reader that it was not possible on time grounds to measure the scattering intensities at 1220 mK for $q_\perp = 0$ whose absence affects the symmetry of Supplementary Fig. 2. The intensities at all energy transfers between 0 and 100 μeV are dominated by elastic scattering and were excluded from our analysis. From a detailed analysis of the measured intensities in all regions of (q_h, E) with and without

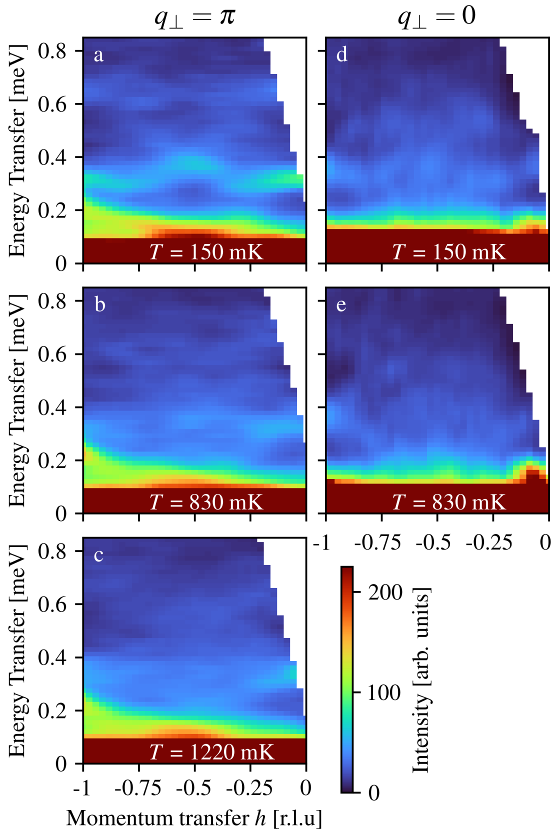

Supplementary Figure 2. **Raw scattering intensity data measured in BPCC at half magnetization for all experimental temperatures. a-c** Neutron scattering intensities measured in sector $q_\perp = \pi$ with $m^z = 0.5$ ($B^z = 2.876$ T) at temperatures of 150, 840 and 1220 mK, shown before background subtraction. **d-e** Analogous intensities measured in sector $q_\perp = 0$ at temperatures of 150 and 840 mK.

Supplementary Table 1. Triplet excitation branches contributing to the different dynamical structure factors defined in Supplementary Eq. (2) and appearing in Supplementary Fig. 5. Because the t^+ and t^- branches appear in the same sectors, we separate their scattered intensity contributions at the median energy of the t^0 continuum.

	S_0^{xx}	S_0^{zz}	S_π^{xx}	S_π^{zz}
branches	t^0	t^+	t^+	t^0
		t^-	t^-	

magnetic scattering, three further contributions to the background were identified: (i) an overall constant; (ii) quasi-elastic scattering appearing at energy transfers up to 200 μeV, which was modelled using a Gaussian with constant width, a Q-dependent amplitude and no dependence on the temperature; (iii) a weak contribution due to neutrons scattered elastically from the magnet, which then arrived at the detectors with a time delay that gave the appearance of a finite energy transfer. The third contribution was modelled using a Gaussian with constant width but with a Q-dependent amplitude, a maximum in E that depended linearly on Q (from $E = 0$ at $q_h = -0.54$ to $E = 0.21$ meV at $q_h = -1$) and again no temperature-dependence. It was ascribed to the sample environment on the grounds that it was not present in symmetry-equivalent Q regions, whereas it did continue to higher energies at $q_h < -1$.

Supplementary Note 3. SUPERSYMMETRY

A. Supersymmetric Quantum Mechanics

To introduce the primary features of a supersymmetric system, we begin[6] with the illustrative example of any Hamiltonian that may be written in the form $H = \frac{1}{2}\{Q, Q^\dagger\}$, where $\{A, B\} = AB + BA$ is the anticommutator. Clearly $[H, Q] = [H, Q^\dagger] = 0$, whence $Q^2 = Q^{\dagger 2} = 0$. The energy of any state is a positive semidefinite quantity, $E \geq 0$. Here we will not dwell on the situation with an $E = 0$ subspace, although this case has additional explicit supersymmetry of the ground state.[6,7]

Most important for our purposes, states with energies $E > 0$ are constrained to come in pairs that are linked by the action of Q and $Q^\dagger$. By defining $c = Q/\sqrt{2E}$, one obtains the standard fermionic algebra $\{c, c^\dagger\} = 1$, whose two-dimensional irreducible representation is spanned by the (degenerate pair of) states $|0\rangle$ and $|1\rangle$. By defining a fermion number operator, $F = c^\dagger c$, one may assign the $|0\rangle$ state of each $E > 0$ multiplet, which satisfies $F|0\rangle = 0$, to the bosonic sector of the Hilbert space and the $|1\rangle$ state ($F|1\rangle = |1\rangle$) to the fermionic sector. This Hilbert space therefore decomposes into the form $\mathcal{H} = \mathcal{H}_B \oplus \mathcal{H}_F$ and we see how every fermionic (bosonic) state has a bosonic (fermionic) counterpart; these superpartners are linked by the supercharge operator, Q, and its conjugate, $Q^\dagger$.

B. Supersymmetric Operators of the t-J Model

To illustrate further consequences of supersymmetry it is convenient to consider explicit examples. We proceed directly to the supersymmetric t-J model,[8–10] which we express in the form

$$H_{tJ} = -t \sum_{i,\sigma} \mathcal{P} c^\dagger_{i,\sigma} c_{i+1,\sigma} \mathcal{P} + H.c. \quad (3)$$
$$+J \sum_i \left(\mathbf{S}_i \cdot \mathbf{S}_{i+1} - \tfrac{1}{4} n_i n_{i+1}\right) + \mu \sum_i n_i,$$

with $J = \mu = 2t$. Here $c^\dagger_{i\sigma}$ ($c_{i\sigma}$) is the creation (annihilation) operator of an electron at site i with spin $\sigma = \tilde{\uparrow}, \tilde{\downarrow}$, $n_i = n_{i\tilde{\uparrow}} + n_{i\tilde{\downarrow}}$ is the electron number operator and $\mathcal{P} = \Pi_i(1 - n_{i\tilde{\uparrow}} n_{i\tilde{\downarrow}})$ is the projector to the Hilbert space of no double site occupancy. We note that the chemical potential, μ, need not be included in the discussion of a general Hamiltonian, but is required for parts of a supersymmetric analysis.

The projector ensures that there are three local states at every site, $|\tilde{\uparrow}\rangle$, $|\tilde{\downarrow}\rangle$ and $|0\rangle$. Nine operators then act in this local Hilbert space, $X^{ab} = |a\rangle\langle b|$ with $a, b \in \{\tilde{\uparrow}, \tilde{\downarrow}, 0\}$, which one may express in the form[10]

$$\begin{aligned}
J_1 &= X^{\tilde{\downarrow}\tilde{\uparrow}} &&= S^- = c^\dagger_{\tilde{\downarrow}} c_{\tilde{\uparrow}}, \\
J_2 &= X^{\tilde{\uparrow}\tilde{\downarrow}} &&= S^+ = c^\dagger_{\tilde{\uparrow}} c_{\tilde{\downarrow}}, \\
J_3 &= \tfrac{1}{2}(X^{\tilde{\uparrow}\tilde{\uparrow}} - X^{\tilde{\downarrow}\tilde{\downarrow}}) &&= S^z = \tfrac{1}{2}(n_{\tilde{\uparrow}} - n_{\tilde{\downarrow}}), \\
J_4 &= X^{0\tilde{\uparrow}} &&= Q_{\tilde{\uparrow}} = (1 - n_{\tilde{\downarrow}})\, c_{\tilde{\uparrow}}, \\
J_5 &= X^{\tilde{\uparrow}0} &&= Q^\dagger_{\tilde{\uparrow}} = (1 - n_{\tilde{\downarrow}})\, c^\dagger_{\tilde{\uparrow}}, \\
J_6 &= X^{0\tilde{\downarrow}} &&= Q_{\tilde{\downarrow}} = (1 - n_{\tilde{\uparrow}})\, c_{\tilde{\downarrow}}, \\
J_7 &= X^{\tilde{\downarrow}0} &&= Q^\dagger_{\tilde{\downarrow}} = (1 - n_{\tilde{\uparrow}})\, c^\dagger_{\tilde{\downarrow}}, \\
J_8 &= X^{00} + \tfrac{1}{2}(X^{\tilde{\uparrow}\tilde{\uparrow}} + X^{\tilde{\downarrow}\tilde{\downarrow}}) &&= T = \mathbb{I} - \tfrac{1}{2}(n_{\tilde{\uparrow}} + n_{\tilde{\downarrow}}), \\
J_9 &= X^{00} + X^{\tilde{\uparrow}\tilde{\uparrow}} + X^{\tilde{\downarrow}\tilde{\downarrow}} &&= \mathbb{I}.
\end{aligned}$$

In an extended system these operators carry an additional site index ($J_{i,\alpha}$). The supersymmetric t-J Hamiltonian, H_{tJ}, commutes with all of the operators of the extended system: if one defines $J^{\rm tot}_\alpha \equiv \sum_i J_{i,\alpha}$ then

$$[H_{tJ}, J^{\rm tot}_\alpha] = 0. \quad (4)$$

The operators J_1 to J_8 generate the subalgebra su(1|2) of u(1|2). In this Lie superalgebra, the operators J_4 to J_7 are fermionic, while the others are bosonic. The operators J_1 to J_3 and J_8 (and trivially J_9) are expected, based on the SU(2) spin symmetry and the charge (hole number) conservation of the t-J model, but the fact that the operators J_4 to J_7 commute with the Hamiltonian is unexpected, and this is what constitutes the supersymmetry. We remark that the t-J model on an extended system [Supplementary Eq. (3)] has a structure more complex than the explicitly supersymmetric form $H = \frac{1}{2}\{Q, Q^\dagger\}$ of Supplementary Note 3A, but shares the key property of paired bosonic and fermionic states. We stress again two points made in the main text. First, in a strict sense the Heisenberg t-J model has two supersymmetries, J_4 and J_5 relating the $|\tilde{\uparrow}\rangle$ fermion to the boson and J_6 and J_7 relating the $|\tilde{\downarrow}\rangle$ fermion to the boson. In the ladder-derived t-J model, as we show next, only one of these supersymmetries is preserved. Second, the supersymmetry applies to the $J^{\rm tot}_\alpha$ operators of the extended system, and the sum over all lattice sites restricts supersymmetric effects to $\mathbf{q} = \mathbf{0}$ in reciprocal space.

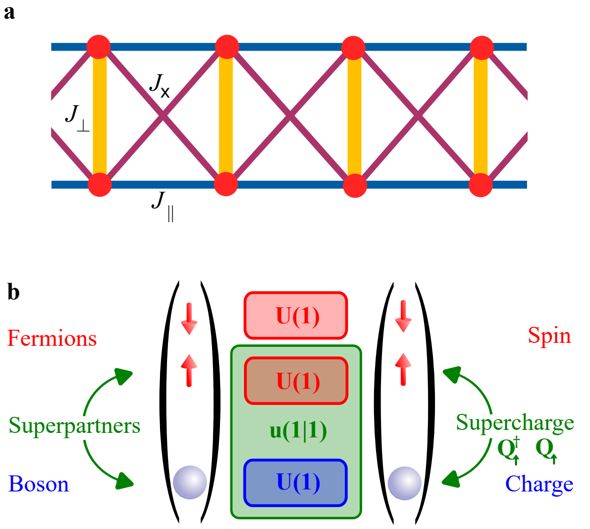

Supplementary Figure 3. **General Heisenberg spin-ladder model.** **a**, Geometry of a two-leg spin ladder. Red spheres represent $S = 1/2$ degrees of freedom on every site and solid lines represent Heisenberg interactions between spin pairs. In addition to the rung ($J_\perp$) and leg ($J_\parallel$) couplings we include a diagonal coupling, $J_\times$, that is absent in the ladder materials BPCB and BPCC, but has an important qualitative effect on the symmetry of the effective hopping terms for $\tilde{\uparrow}$ and $\tilde{\downarrow}$ holes in the t-J-model description of the t^0 excitation branch. **b**, Representation of supersymmetry in the ladder-derived t-J-chain model. The extended symmetry of the three site-basis states takes the form that the symmetry of the fermionic spins is U(1) and only the up-spin species has an additional duality with the bosonic hole state, making these superpartners with corresponding supercharge operators $Q_{\tilde{\uparrow}}$ and $Q_{\tilde{\uparrow}}^\dagger$.

Here we do not comment further on the relationships enforced by supersymmetry on the energy spectra of sectors with different particle numbers, other than to note that these extend well beyond the zero- and one-hole sectors we consider.[9] In the spin ladder, the way in which the magnetic field functions as an effective spinon chemical potential has already been used extensively in describing the low-energy response, and we review this in Supplementary Note 4. The nontrivial role of the chemical potential in supersymmetric phenomena suggests a further avenue for experimental exploration, through the effect of the applied magnetic field.

C. Ladder-derived t-J model

To review the mapping of the Heisenberg spin ladder in an applied magnetic field to an effective chain model, we extend the original derivation[11] by considering the more general ladder geometry of Supplementary Fig. 3a. We note that more general interactions leading to integrable ladder models are reviewed in Supplementary Ref.[12] Restricting our considerations to Heisenberg interactions, the two diagonal couplings within $J_\times$ need not be symmetric, and have a qualitative effect on the dynamics of the chain model. The mapping is a strong-coupling expansion, which makes use of the fact that $J_\perp \gg J_\parallel$ in Eq. (1) of the main text. In the low-energy sector, meaning that no rungs are excited, the two allowed rung states, $|\tilde{\downarrow}\rangle \equiv |s\rangle$ and $|\tilde{\uparrow}\rangle = |t^+\rangle$, form a pseudospin-1/2 basis with effective Hamiltonian

$$H_{\rm XXZ} = \sum_i \left[J_{xy}(S_i^x S_{i+1}^x + S_i^y S_{i+1}^y) + J_z S_i^z S_{i+1}^z\right] \\ -h\sum_i S_i^z + \text{const.}, \tag{5}$$

where

$$J_{xy} = J_\parallel - J_\times, \qquad J_z = \tfrac{1}{2}(J_\parallel + J_\times) \tag{6}$$

and

$$h = b^z - J_\perp - \tfrac{1}{2}(J_\parallel + J_\times). \tag{7}$$

This effective XXZ spin Hamiltonian has the well known anisotropy $\Delta = J_z/J_{xy} = 1/2$ when $J_\times = 0$, placing the spin chain in the Luttinger-liquid universality class.

Excitations of the $J_\perp$ energy sector of the ladder, meaning those of a single t^0 triplet, create a hole in the pseudospin background, $|0\rangle \equiv |t^0\rangle$. Hence they can be described precisely by the states of a single hole in an otherwise half-filled, one-dimensional t-J model with Hamiltonian

$$H_{tJI} = t_{\tilde{\uparrow}}\sum_i (c_{i\tilde{\uparrow}}^\dagger c_{i+1\tilde{\uparrow}} + \text{H.c.}) + t_{\tilde{\downarrow}}\sum_i (c_{i\tilde{\downarrow}}^\dagger c_{i+1\tilde{\downarrow}} + \text{H.c.}) \\ +J_{\rm I}\sum_i (n_{ih}n_{i+1} + n_i n_{i+1h}) \\ +\mu\sum_i n_{ih} + H_{\rm XXZ}, \tag{8}$$

where

$$t_{\tilde{\uparrow}} = \tfrac{1}{2}(J_\parallel + J_\times), \qquad t_{\tilde{\downarrow}} = \tfrac{1}{2}(J_\parallel - J_\times), \\ J_{\rm I} = -\tfrac{1}{4}(J_\parallel + J_\times) \ \text{and} \ \mu = \tfrac{1}{2}(b^z + J_\perp). \tag{9}$$

Here $c_{i\sigma}^\dagger$ and $c_{i\sigma}$ are respectively the creation and annihilation operators of a fermion at site i with pseudospin $\sigma = \tilde{\uparrow}, \tilde{\downarrow}$, n_i is their number operator on site i and n_{ih} is the number operator for the presence of a hole on site i. The $t_{\tilde{\uparrow}}$ and $t_{\tilde{\downarrow}}$ terms express how the fermion hopping terms are split by a finite $J_\times$ and the term $J_{\rm I}$ is a nearest-neighbour interaction that acts only between the hole and the $\tilde{\uparrow}$ pseudospins.

By following this mapping, one observes the direct correspondence between the t_0 branch of the spectral function $S_{q,0}^{+-}$ of the ladder and the single-particle spectral function of the pseudospin-up holes of the equivalent chain model, $\langle c_{q\tilde{\uparrow}}^\dagger c_{-q\tilde{\uparrow}}\rangle$, where

$$\langle c_{q\sigma}^\dagger c_{-q\sigma}\rangle = \sum_i \int dt\, e^{+i(\omega t - q x_i)} \langle c_{i\sigma}^\dagger(t) c_{0\sigma}(0)\rangle. \tag{10}$$

Similarly, the t_0 branch of the $S^{zz}_{q,\pi}$ spectral function is reproduced directly by the single-particle spectral function of the pseudospin-down holes, $\langle c^{\dagger}_{q\tilde{\downarrow}} c_{-q\tilde{\downarrow}} \rangle$. We stress again that the Heisenberg origin of the starting ladder guarantees the presence of one pole in the spectral function, and we will show explicitly in Supplementary Note 6 that it appears in $\langle c^{\dagger}_{0\tilde{\uparrow}} c_{0\tilde{\uparrow}} \rangle$ but not in $\langle c^{\dagger}_{0\tilde{\downarrow}} c_{0\tilde{\downarrow}} \rangle$.

In the context of supersymmetry, this pole is the expression in the $\mathbf{q} = \mathbf{0}$ momentum sector of the duality between the bosonic holes and the fermionic $\tilde{\uparrow}$ spins. In the terminology of Supplementary Note 3B, J_4 and J_5 are still valid supercharge operators in the ladder-derived t-J model, but J_6 and J_7 are not. This situation is depicted in Supplementary Fig. 3b, where the SU(2) symmetry of the effective spin sector (present in Fig. 1f of the main text) is broken by the XXZ nature of the effective Hamiltonian [Supplementary Eq. (5)]. We stress that the remaining supersymmetry persists even in a situation where strong $J_{\times}$ ensures a significant asymmetry in the hopping and interaction parameters of the two spin species.

To study the spectral functions of the ladder-derived t-J model at finite temperatures, the fact that the model is well defined only for zero or one hole in the chain constrains our considerations to the limits $T \ll b$ or $T \ll J_{\perp}$, whereas $T/J_{\parallel}$ can be large. Thus we work effectively in a limit where the chemical potential for a single hole satisfies $\mu \gg T$ and we need to include only the XXZ spin sector in the thermal density matrix, while the hole sectors are not thermally populated. In this limit we compute the spectral functions for the removal of $\tilde{\uparrow}$ and $\tilde{\downarrow}$ electrons, hence creating holes of two different origins; these are the quantities shown in Figs. 4 and 5 of the main text at different momentum transfers and temperatures, and in Supplementary Note 6 we compare the full zero-temperature spectral functions in the two cases.

We close by discussing the statistics of all the quasiparticles in these models. In the ladder-derived t-J model, all entities are bosonic, precisely because it is derived from a pure spin model. However, performing a Jordan-Wigner transformation on the spin species and exchanging the sign of the hopping term, t, constitutes a unitary mapping of the ladder-derived t-J model to an anisotropic version of a single-chain FFB model, with two fermionic and one bosonic species. The location in momentum space of the supersymmetry-related δ-function is not changed under this mapping.

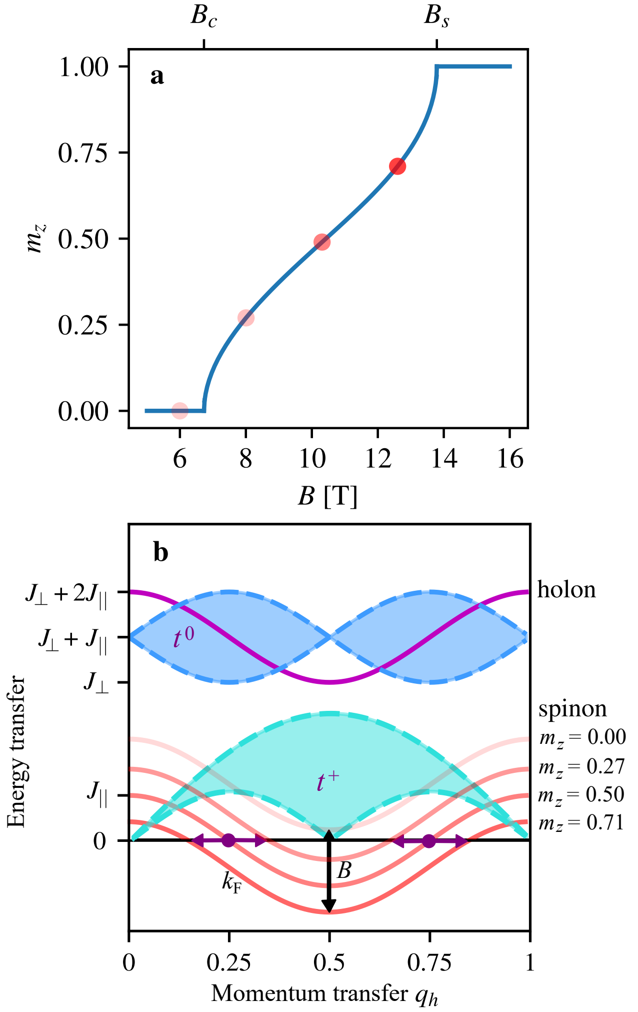

Supplementary Figure 4. **Two-leg ladder in a magnetic field.** **a** Magnetization of a two-leg ladder at zero temperature, calculated using l-MPS (solid blue line) for the parameters of BPCB, where B_c is 6.9 T and B_s is 13.8 T. The red points show the field values used in INS experiment performed on ThALES. In BPCC, $B_c = 1.6$ T and $B_s = 4.07$ T. **b** Excitations of the t^+ (dCPF, turquoise) and t^0 (blue) sectors at half magnetization, as depicted in Fig. 1c of the main text. The red line labelled $m^z = 0.5$ marks the cosine dispersion of an effective spinon at half magnetization, two of which combine to produce the dCPF continuum. The lighter and darker red lines indicate how the effective Fermi wavevector, $k_F = \pm\pi/2$ at $m^z = 0.5$, is altered by the field. The violet cosine marks the effective dispersion of a single bare t^0 state, or hole, whose convolution with one spinon at k_F produces the blue continuum that is described by an adapted t-J model.

Supplementary Note 4. SPIN-LADDER PHYSICS

A. Spin-1/2 two-leg ladder in a magnetic field

We summarize the properties of the two-leg $S = 1/2$ ladder in an applied magnetic field, as described by Eq. (1) of the main text. Previous neutron spectroscopy studies[2,3] have fixed all the interaction parameters of BPCB and BPCC to high accuracy. From the values quoted in the main text, both materials are strong-rung ladders ($J_{\perp} \gg J_{\parallel}$) and thus applied fields on the order of $J_{\perp}$ allow a clear separation of the different spin excitation branches. The field commutes with the Hamiltonian and at low fields causes a rigid energy shift (Zeeman splitting) of the three triplet branches, t^+, t^0 and t^-, without changing their shape. Figure 1b of the main text repre-

sents the locations of these bands as the field is increased. When $B^z < B_c$, the gapped ground state is dominated by rung singlets ($|s\rangle$), and when $B^z \geq B_s$ (the fully polarized regime) it is dominated by rung triplets ($|t^+\rangle$); in both cases, the spin excitations are $\Delta S = 1$ entities whose discrete "triplon" branches have been studied in detail.[3]

By contrast, when $B_c \leq B^z \leq B_s$ the ground state is partially magnetized (Supplementary Fig. 4a) and can be expressed as a coherent superposition of $|s\rangle$ and $|t^+\rangle$ rungs. The spin excitations become gapless and the spin degrees of freedom fractionalize into pairs of spinons, effectively $\Delta S = 1/2$ entities. Their deconfined nature in this field range ensures the continuum response functions measured for all three branches in Figs. 2b-d, 3a and 3e of the main text. The low-energy physics of the gapless (t^+) branch can be described in the framework of a TLL whose Fermi points, $k_{\rm F} = \pi(1 \pm m^z)$, have a linear dependence on the ladder magnetization (an arccosine dependence on the scaled B^z), as represented in Supplementary Fig. 4b. The physics of the gapped t^0 branch is the focus of our present contribution, and the strong rung coupling ensures that the t^+ and t^0 continua remain mostly non-overlapping at all temperatures. A consistent description of the full field-dependent spectrum would interpret the gapped regime in terms of confined spinons and the field-driven quantum phase transitions (QPTs) at B_c and B_s as confinement-deconfinement processes.

B. Spin Luttinger Liquid

Here we summarize some of our results for the t^+ sector, which lie outside our present focus but are nonetheless of value to the overall understanding of spinon physics. For fields between B_c and B_s, the $\Delta S = 1$ excitation spectrum is described rather accurately by the dCPF continuum deduced[13] for fully deconfined spinons. Theoretically, a complete account of the t^+ spectrum at $T = 0$ has been sought by Bethe-Ansatz[14,15] and DMRG[11] calculations. The TLL framework, which approximates the spectrum at the lowest energies by bosonization of its linear lower edges, has been used to describe ladder thermodynamic properties, which are dominated by the gapless points.[16] The fact that the ladder has two symmetry sectors, $q_\perp = 0$ and $q_\perp = \pi$, means that there are in fact two dCPF continua, visible at low energies in Figs. 3c and 3g of the main text. Unlike the analogous t^0 sectors, these show no differences in energy, i.e. the support is identical in both cases. However, the intensities and their thermal evolution differ significantly.

Because the t^+ continuum is obscured by the elastic line below 0.5 meV in our BPCB experiment and 0.15 meV in our BPCC measurement, an experimental study of the t^+ continuum was not possible here, but was performed for BPCB in Supplementary Ref.[14] In the t^+ spectral weight computed by MPS for $q_\perp = \pi$ (Fig. 3c of the main text), the characteristic dCPF support has strong intensity along its lower edge becoming maximal at $q_h = -0.5$. At $q_\perp = 0$ (Fig. 3g), the peak intensities are significantly lower and there is a distinctly more uniform distribution of spectral weight across the continuum. This difference arises from the fact that the spectrum at $q_\perp = \pi$ measures transverse correlations (S^{+-}_π), while that at $q_\perp = 0$ is longitudinal (S^{zz}_0), and the two have different phase-space criteria for two-spinon contributions.

At finite temperatures, the MPS t^+ continua do not develop a gap, but the maximum in spectral weight does shift for $q_\perp = \pi$ to a finite energy of the same order as the temperature (Figs. 3d and 4b of the main text). This upward weight distribution at $q_h = -0.5$ contrasts with a downward one around $q_h = -0.25$ (Fig. 3d). The thermal evolution at $q_\perp = 0$ is quite different, with a generalized shift of intensity from the bottom to the top of the continuum over a wide range of q_h (Fig. 3h), which around $q_h = -0.5$ constitutes a dramatic shift of the maximum far in excess of the value of T (Fig. 4d). While the TLL framework can be used to describe the shift of maximum spectral weight in the $q_\perp = \pi$ continuum, it does not account for the much stronger shift when $q_\perp = 0$.[17] Despite the strong redistribution of spectral weight within each dCPF continuum, other than at $q_h = 0$, there is no discernible thermally induced change in their position. We comment that the finite-T t^+ continuum has been measured in the spin-chain material $KCuCl_3$ and the results analysed by MPS methods,[15] but the energy scales in this compound make it unsuitable for the controlled application of laboratory magnetic fields.

The excitation spectra shown in Fig. 3 of the main text as functions of q_h were obtained by integration over all values of the transverse momenta, because all modes are non-dispersive for out-of-plane scattering, and over a window of width $\Delta l = 0.25$, with $l = [n + 1/2 - 0.3822 q_h]/0.4866$ and $n = 0$ (0.5) for $q_\perp = \pi$ (0). These integrations were performed using a modified version of the Horace toolbox.[18] The data were then rebinned and a five-point moving-average filter was applied. Elastic scattering contributions were modelled by a Gaussian function with constant width but a **Q**-dependent amplitude. Multiple scattering events from the sample environment were dominated by the cryomagnet and were modelled by a second Gaussian with a **Q**-dependent energy offset of less than 0.26 meV and a width of up to 0.44 meV. The remaining background, arising from detector noise and incoherent phonon scattering, was modelled as a constant. The sum of these contributions, the quantity to which we refer as "the background," was modelled individually for each integration range and was kept fixed for all temperatures.

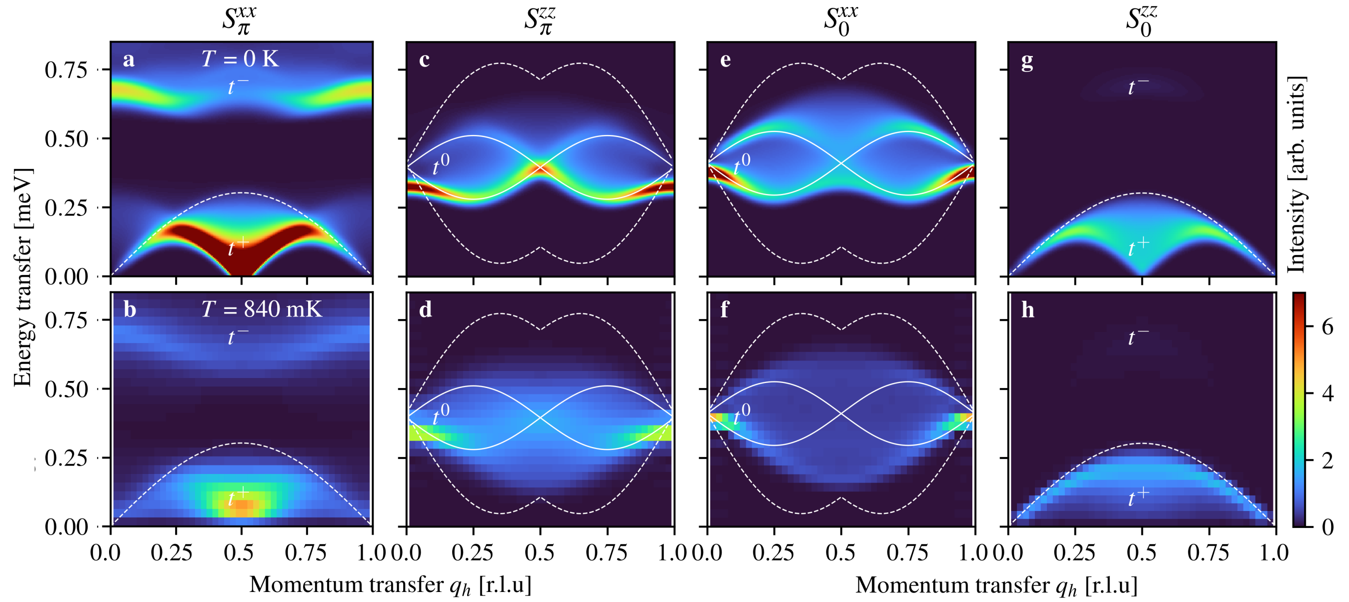

Supplementary Figure 5. **Dynamical spectral functions calculated by MPS for the parameters of BPCC at $m^z = 0.5$.** S_π^{xx} (a,b), S_π^{zz} (c,d), S_0^{xx} (e,f) and S_0^{zz} (g,h) are shown at temperatures $T = 0$ (a,c,e,g) and 840 mK (b,d,f,h). As in Fig. 3 of the main text, the separate functions are weighted by the kinematic and structure factors of INS given in Supplementary Eq. (1). Solid lines show the zero-field t^0 dispersions shifted by $k_{\rm F} = \pm\pi/2$ that form the basis of the t-J-model description of the t^0 continuum (main text). Dashed lines in panels a, b, g and h mark the upper edge of the dCPF continuum and in panels c-f they mark the outer limits to the nominal support of three-spinon t^0 processes.

Supplementary Note 5. LADDER MPS SPECTRAL FUNCTIONS

MPS methods are variational approaches that approximate the required state or density matrix within the space of MPS with a maximal matrix dimension (bond dimension) of χ.[19] It has been demonstrated that MPS methods provide a very accurate account of the physical properties of low-dimensional quantum spin systems, their numerical accuracy being ensured by adjusting χ and monitoring the truncation error, which quantifies the neglected weight in the required density matrix.

We performed MPS calculations for two different purposes, namely investigating the intrinsic properties of ladder and chain spectra and modelling the spectral functions measured by INS, which sometimes required different specifications. The nature of these calculations depended strongly on whether they were performed at zero or finite temperature. At zero temperature we used ladders with sizes up to 2×200 sites. At finite temperatures, where the shorter correlation lengths justify the use of shorter systems, we used a size of 2×45 sites. Spectral functions in an applied magnetic field were computed by setting the total spin to S^z, meaning that they were performed inside a specific magnetization sector, $m^z = S^z/2L$, where L denotes the number of ladder rungs. The conversion from m^z to the applied field, B^z, used for our BPCB experiments in Fig. 2 of the main text, was made using the magnetization calculated at zero temperature in Supplementary Ref.[11] The time steps used to achieve convergence in our finite-T calculations were $\Delta t = 0.0625\hbar/J_\perp$ and $\Delta\tau = 0.02\hbar/J_\perp$, with a bond dimension of $\chi = 620$ ensuring a total truncation error below 10^{-2}, or a single-step truncation error below 10^{-5} at the final time, $t_{\rm max}$.[17] At $T = 0$, $\Delta t = 0.1\hbar/J_\perp$ and $\chi = 200$ were sufficient to obtain well converged results in Supplementary Fig. 5. We defer to Supplementary Note 6 the specifications of the calculations used in the preparation of all the figures in the main text.

A. Separating ladder spectral functions by spin sector

Here we show the individual dynamical spectral functions, $S_{q_\perp}^{xx}$ and $S_{q_\perp}^{zz}$ of Eqs. (1) and (2), calculated by ladder MPS at low and intermediate temperatures in both the antisymmetric and symmetric parity sectors. This allows a direct visualization of the separate spectral contributions in Table 1, and hence of the thermal effects on scattering in the separate triplet continua. Supplementary Figs. 5a-b and 5c-d show respectively S_π^{xx} and S_π^{zz} at $T = 0$ and 840 mK; S_π^{xx} contains excitations from $|s\rangle$ to $|t^+\rangle$ and $|t^-\rangle$ and S_π^{zz} from $|s\rangle$ to $|t^0\rangle$. Supplementary Figs. 5e-f and 5g-h show respectively S_0^{xx} and S_0^{zz} at $T = 0$ and 840 mK, S_0^{xx} containing excitations from $|t^+\rangle$ to $|t^0\rangle$ and S_0^{zz} from $|t^+\rangle$ to $|t^+\rangle$ and $|t^-\rangle$.

We emphasize three qualitative points. First, we are considering thermal evolution over the range from $T = 0$ to $T \approx J_\parallel$. Although one might expect that the spinon

excitations within each triplet branch would begin to lose coherence at temperatures of this order, it is clear from the observed spectra that a spinon interpretation remains valid at least to the highest temperatures we consider. Second, as described in Supplementary Note 3B, comparing the t^+ sectors (Figs. 5a-b with Figs. 5g-h) makes clear that the average spectral weight of the symmetric continuum is lower in intensity, higher-lying in energy and displays a much stronger thermal shift of weight to higher energies, visible in Figs. 4c-d of the main text. Third, the thermal evolution in both t^0 sectors (Supplementary Figs. 5c-d and 5e-f) illustrates the role of additional multispinon scattering processes that become visible due to the thermal occupation of initial states, as we describe next.

B. t^0 continua

We have shown how INS measurements of the t^0 spectrum provide a clean experimental "quantum simulation" of the spectral response of a t-J chain with none of the complexities of eV charge energies typical in correlated electronic materials. Nevertheless, the two-leg spin ladder has two t^0 continua, for the symmetric and antisymmetric parity sectors, and the situation extends beyond that of the pure t-J chain[20–22] in two respects, namely the different interaction terms (Supplementary Note 1C) and the possibility of controlling the chain magnetization with an applied magnetic field. As also noted in the main text, thermal effects on the two t^0 continua are highly non-trivial both by type and extent of the observed weight shifts and by differences across the Brillouin zone. In Fig. 4 of the main text, we integrated the scattered intensity over a range of wavevectors around $q_h = -0.5$ (conventionally the Brillouin zone edges), in Fig. 5 of the main text around $q_h = 0$ or -1 (zone centre), and in Fig. 6 we integrate around $q_h = -0.75$ (mid-zone). Here we use this division to describe a number of additional effects that are observable in the integrated data.

Zone centre. In our low-temperature measurements we observe rather compact spectra (Figs. 3a,c,e,g and Figs. 5c,e of the main text) with the strong lower edges lying at 0.32 meV for $q_\perp = \pi$ but 0.38 for $q_\perp = 0$; these values are interchanged from the positions of the lower edges at $q_h = -0.5$. This difference could already be inferred from the fact that the spectral function of the Heisenberg model in an applied field contains one exact pole and not two. Although the effects of finite resolution in our INS measurement and finite system size in our MPS calculations mean that it is not possible to deduce the nature of the pole in the $q_\perp = 0$ channel directly, it is an important physical result that the continuum response (i) diverges at the band centre and (ii) exhausts the spectral weight there, preventing the separation of a(n anti-)bound state. True bound-state behaviour in the unfrustrated two-leg ladder appears to be restricted to the limits of zero and full polarization by the applied

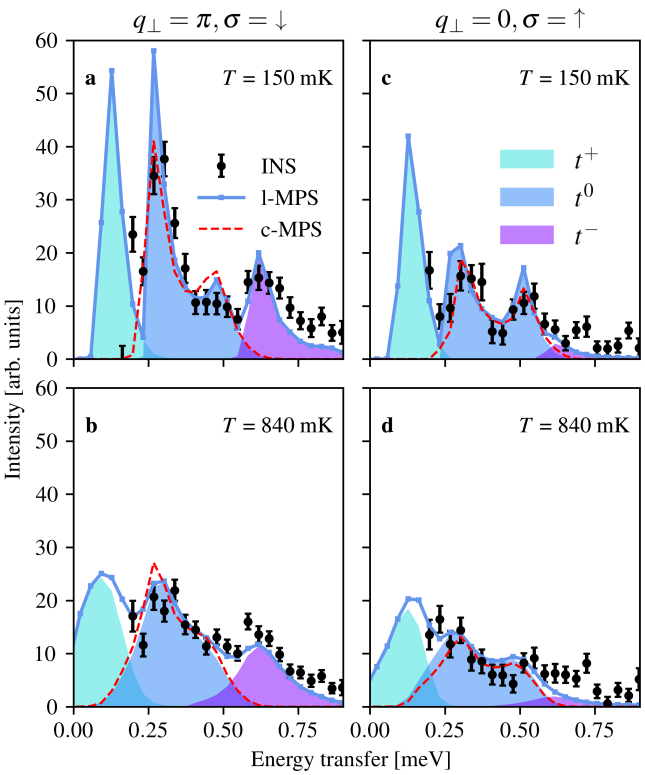

Supplementary Figure 6. **Mid-zone many-body thermal broadening.** Background-subtracted scattered intensities measured for BPCC at $m^z = 0.5$ and at low (a,c) and intermediate (b,d) temperatures, integrated over momentum transfers $q_h \in [-0.875, -0.625]$ and shown for sectors $q_\perp = \pi$ (a,b) and $q_\perp = 0$ (c,d). The error bars indicate one standard deviation.The solid blue line is the spectral function of a two-leg spin ladder and the dashed red line the spectral function of a single hole in a t-J chain, both computed by zero-temperature MPS in the upper row and by finite-temperature MPS in the lower row.

field,[23] i.e. $m^z \to 0$ and $m \to m^z_{\mathrm{sat}}$, and hence to occur only far from our present focus on $m^z = 1/2$.

Turning to finite temperatures, quite in contrast to the behaviour of the t^0 continua elsewhere in the Brillouin zone, there is in essence no shift of spectral weight at $q_h = 0$ (Supplementary Figs. 5d,f). For $q_\perp = \pi$, the strong lower boundary of the continuum remains fixed at approximately 0.32 meV, with the effect of temperature being restricted to a rather conventional broadening (Fig. 5c of the main text). This is a kinematic effect that results from the vanishing phase space for additional spinon-pair processes at this wavevector. By contrast, the fact that the lower boundary of the continuum is fixed at 0.38 meV for $q_\perp = 0$ is a rigorous consequence of supersymmetry.

Mid-zone. For wavevectors around $q_h = -0.75$, the t_0 continua have their maximum bandwidth. Supplementary Figs. 6a,c show a significant difference in the spectral weights at the lower and upper edges, with the stronger upper edge in the $q_\perp = 0$ sector reminiscent of the situa-

tion in the t^+ continuum. As the temperature is raised, the leading effect in both parity sectors is the suppression of both peaks (Supplementary Figs. 6b,d), while in Supplementary Figs. 5d,f one observes more clearly that this is accompanied by a downward shift of spectral weight. The mechanism for this shift in terms of thermal occupation of two-spinon states is explained next for the case of $q_h = -0.5$, where its extent in energy is much greater.

Zone edge. As noted elsewhere, the low-T spectrum at $q_h = -0.5$ narrows to a rather sharp "knot of the bow-tie" feature with its onset at 0.32 meV for $q_\perp = 0$ but 0.38 meV for $q_\perp = 0$. The concentration can be understood from the straightforward convolution of a noninteracting spinon and holon,[24] and the difference between sectors from interaction effects. The most dramatic thermal effect in our entire investigation is the downward broadening of the knot to cover a giant energy range from 0.15 up to 0.35 meV ((Supplementary Figs. 5d,f), whereas its upward extent changes rather little. With energies below $\omega = 0.25$ meV forbidden in the t^0 continuum at $T = 0$, this phenomenon requires a many-body explanation.

At finite temperatures, the bare t^0 holon can be dressed by multispinon processes of all odd orders, and exact results for one-holon–one-spinon and one-holon–three-spinon excitations were obtained in Supplementary Ref.[25] The leading corrections to the t^0 spectrum are scattering processes involving three spinons, which physically involve holon-spinon separation together with a single spin-flip. Thus the continuum includes processes where the holon dispersion (Supplementary Fig. 4) is shifted in momentum by three units of $\pm\pi/2$ (that still sum to $\pm\pi/2$) and in energy by all possible energies of the additional spinon pair. The qualitative result is to extend the support in momentum and energy by adding a (two-spinon) dCPF continuum above the t-J (one-spinon) response, i.e. at positive energy transfer, and another continuum below it at negative transfer, as depicted by the dashed lines in Supplementary Figs. 5c-f. Processes in the positive continuum are allowed at $T = 0$ and indeed are visible in Figs. 3c,g of the main text[11] (shown more clearly in Supplementary Figs. 5c,e). The qualitative change at finite T is to occupy additional initial states,[25] making it possible to observe an inverted dCPF continuum lying below the $T = 0$ t^0 spectrum, as Supplementary Figs. 5d,f make clear. This accounts directly for the observation in Figs. 4b,d of the main text of strong spectral weight in the range $\omega = 0.15$-0.3 meV at $q_h = -0.5$.

For completeness, we conclude by drawing attention to the thermally induced changes in the t^- continua. Although less clear in experiment (Figs. 3a,e of the main text), these are well resolved by the MPS calculations shown in Supplementary Figs. 5a-b. Here we also observe spectral-weight distributions that are concentrated along the lower edge of the rather narrow continuum at low temperatures, then become more uniform with increasing temperature and also extend towards significantly lower energies around $q_h = -0.5$.

Supplementary Note 6. CHAIN MPS SPECTRAL FUNCTIONS

Our MPS calculations for different t-J models were also performed for chains of lengths up to $L = 200$ sites at zero temperature, while at finite temperatures we used a chain of 40 sites. The total magnetization in the ground state of the chain, $\tilde{S}^z$, was determined from that of the equivalent ladder as $\tilde{S}^z = S^z - L/2$, i.e. again the calculations were performed in a specific magnetization sector; the process of matching m^z as closely as possible to the field values applied in our INS experiments also entailed a small, rigid shift of the spectrum in energy. The number of holes in the chain was $n_h = 0$ in the ground state and $n_h = 1$ while measuring correlation functions. Because the ladder-derived t-J chain is a bosonic model restricted to the zero- and one-hole sectors, the one-hole correlation functions are equivalent to those of a fermionic t-J chain under a momentum shift of π. The equivalence of the chain correlation functions to the correlations obtained at first order in the perturbative expansion of the ladder Hamiltonian is given by

$$S_\pi^{zzz} = \langle c_{\tilde{\downarrow}}^\dagger c_{\tilde{\downarrow}} \rangle,$$
$$S_0^{+-} = 2\langle c_{\tilde{\uparrow}}^\dagger c_{\tilde{\uparrow}} \rangle,$$
$$S_0^{xx} = \tfrac{1}{2}\langle c_{\tilde{\uparrow}}^\dagger c_{\tilde{\uparrow}} \rangle,$$

and small discrepancies between the spectra we compute have their origin in higher-order processes that are allowed in the ladder but are not present in the chain.

In our MPS calculations for the t-J chain, we used a time step $\Delta t = 0.01\hbar/J_\perp$ at $T = 0$, while at finite T the steps were $\Delta t = 0.02\hbar/J_\perp$ and $\Delta\tau = 0.2\hbar/J_\perp$ (respectively for real and imaginary times). By using bond dimensions up to $\chi = 4000$ and keeping the truncation errors below 10^{-10}, we reached final times of approximately $t_{\max} = 80\hbar/J_\perp$ at $T = 0$ and $t_{\max} = 30\hbar/J_\perp$ at finite T. These data were extended in time by employing the method of linear prediction,[26–28] which extrapolates the data for each momentum by effectively fitting to a superposition of exponentially damped and oscillating terms in time. In this way we extended $t_{\max}$ by a factor of 5 at $T = 0$ and a factor of 8 at finite T, achieving respective final times of $400\hbar/J_\perp$ and $240\hbar/J_\perp$.

Here we use our MPS calculations to supplement the discussion of the main text in two aspects. The first is to provide a systematic presentation of the results shown in Fig. 5 of the main text, where the supersymmetric properties of t-J-chain models were inspected by computing the thermal evolution of the $\mathbf{q} = \mathbf{0}$ spectral function. We also show the spectral functions of three different models that make the connection between the pure t-J chain and the model [Supplementary Eq. (8)] for which the two-leg ladder serves as a quantum simulator. The second is to detail the procedures by which our chain-MPS calculations were matched quantitatively to the experimentally measured spectral functions in the t^0 sector.

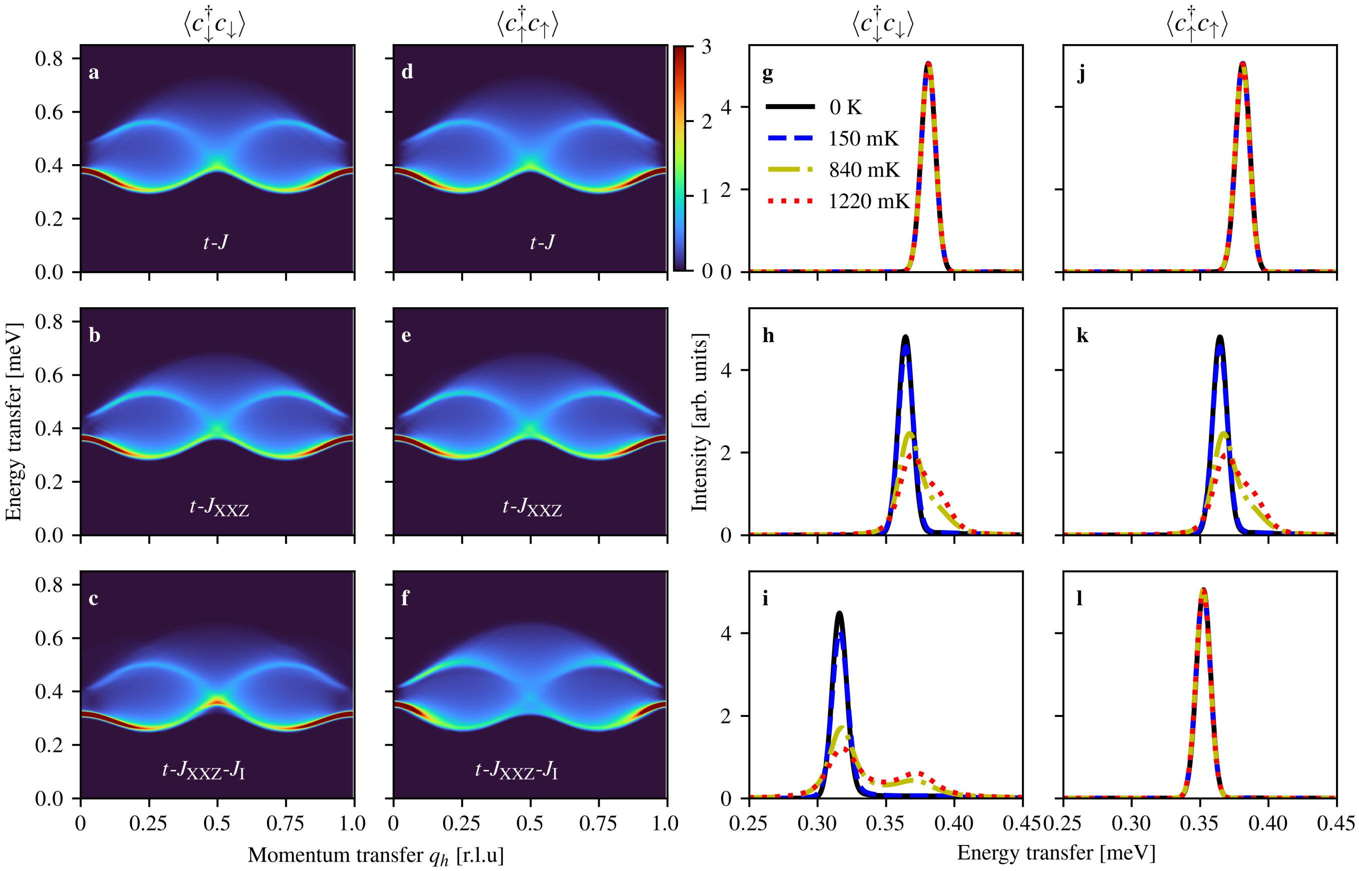

Supplementary Figure 7. **Spectral functions of different *t*-*J* models. a-c** $S(q_h, \omega)$ at $T = 0$ for the down-spin holons of the t-J model (a), the t-J_{XXZ} model with $J_z = J/2$ (b) and the t-J_{XXZ}-J_{I} model (c). **d-f** $S(q_h, \omega)$ at $T = 0$ for the up-spin holons of the t-J model (d), the t-J_{XXZ} model with $J_z = J/2$ (e) and the t-J_{XXZ}-J_{I} model (f). **g-i** Thermal evolution of the down-spin spectral functions for all three models, shown at $q_h = 0$. **j-l** Thermal evolution of the corresponding up-spin spectral functions.

A. Supersymmetric thermal evolution

The minimal t-J-chain model, H_{tJ} in Supplementary Eq. (3), consists only of a single hole-hopping term and a Heisenberg interaction between adjacent spins. As noted in the main text, one nontrivial difference from the ladder-derived t-J model is that the spin interactions of the latter are those of an XXZ model with $J_z = J_\parallel/2$. In Supplementary Figs. 7a-b and 7d-e we compare the excitation spectra of this XXZ model for both spin species (equivalent to both ladder parity sectors) with those of the Heisenberg t-J chain. It is no surprise that the quantitative differences are not large: the XXZ model in the XY regime ($J_z < J_{xy}$) shares the spinon physics of the Heisenberg chain, and the effects of dressing a single hole remain the same for both models and both spin species.

As shown in Supplementary Note 3C, the ladder-derived t-J model differs in one further respect: in the absence of the hopping anisotropy produced by $J_\times$, this is the interaction term (J_{I}) arising between the introduced hole and the neighbouring local spin states of only one species (up-spins). For numerical purposes, this term may be regarded as an advantage, in that it distinguishes between holes of up- and down-spin character. In Supplementary Figs. 7c and 7f we illustrate the spectral function of this model, which splits the two spin sectors to restore the physics of the two-leg ladder (Figs. 3c and 3g of the main text).

To visualize directly which of these t-J models possess a supersymmetry, we computed their finite-temperature spectral functions at $q_h = 0$, using linear prediction to achieve our maximum final times and applying a weak Gaussian filter ($M(x,t)$ in the Methods section) in time only, with filter width $t_{\mathrm{w}} = 80\hbar/J_\perp$. The resolutions of these calculations then took the approximate values, 0.012 meV in energy and 0.025 r.l.u. in momentum, quoted in the main text. Supplementary Figs. 7g,j, which are reproduced in Figs. 5e,j of the main text, show that the δ-function nature of the response is preserved at finite T for both spin species in the pure t-J chain, reflecting the double supersymmetry of this special case (Supplementary Note 3B). By contrast, the t-J_{XXZ} model has no supersymmetries and in Supplementary Figs. 7h,k we observe that neither response function has a δ-function

form at $q_h = 0$, even at $T = 0$, and at $T > 0$ both show a systematic rise in broadening.

The interaction term acts precisely to compensate the deformation of the spin interactions from Heisenberg to XXZ form, restoring the supersymmetry of one spin species as expected from the presence of the pole in the spectral function of the underlying Heisenberg spin ladder. Supplementary Fig. 7l shows a δ-function in $\langle c^\dagger_{\tilde{\uparrow}} c_{\tilde{\uparrow}} \rangle$ irrespective of T, in contrast to Fig. 7i, where the $\langle c^\dagger_{\tilde{\downarrow}} c_{\tilde{\downarrow}} \rangle$ spectral function is neither a δ-function at $T = 0$ and nor does it preserve its width as T increases (these two panels are reproduced in Fig. 5i of the main text). We comment that these different forms of thermal evolution were observed previously in ladder MPS calculations,[17] but it was not clear whether the ladder-derived t-J model could capture their full extent. The results of Supplementary Figs. 4, 5 and 7 confirm that the t-J model remains a faithful representation of the full ladder model at all temperatures up to $T \approx J_\perp$.

B. Matching ladder and chain calculations to experiment

Here we present the details involved in preparing Figs. 2-5 of the main text, all of which show quantitative comparisons between experimental measurements and MPS calculations. Because the INS data we show for BPCB was obtained at the lowest temperature for a range of applied fields, we performed only zero-temperature MPS on a ladder of $L = 160$ rungs and a t-J chain of $L = 160$ sites. The time steps were $t = 0.126\hbar/J_\perp$ ($\Delta t = 0.01$ K^{-1}) for the ladder and $t = 0.01\hbar/J_\perp$ ($\Delta t \simeq 0.001$ K^{-1}) for the chain. The final times reached were $t_{\max} = 252\hbar/J_\perp$ for the ladder and $t_{\max} = 120\hbar/J_\perp$ for the chain. As noted above, the ladder and chain magnetizations at the fields applied in the experiment were deduced from the ladder MPS calculations of Supplementary Ref.[11] To reproduce the experimental data, we convolved the MPS spectral function with the instrumental resolution function of ThALES, which was approximated by a tilted 2D Gaussian with a FWHM of 0.14 r.l.u in momentum and 0.16 meV in energy, with a tilt angle of $\tan(\alpha) = 0.16$ meV/0.06 r.l.u. We note for clarity that Figs. 2j-l of the main text show only the chain-MPS spectral functions of the down-spin holons (i.e. derived from Supplementary Fig. 7f).

Our INS data for BPCC were obtained only at half magnetization, but for two or three different temperatures. For the parameters of BPCC ($J_\perp = 3.42$ K, $J_\parallel = 1.34$ K, $g = 2.26$[3]), the field establishing $m^z = 0.5$ is $B^z = 2.693$ T [Supplementary Eq. (7)]. However, to compensate for the small but finite base temperature of the experiment, the real magnetic field applied to keep the ladder at half-magnetization was $B^z = 2.876$ T, and taking this into account in our MPS calculations produced an energy shift of approximately 0.02 meV.

For the zero-temperature calculations shown in Figs. 3-5 of the main text, the ladder length was 200 rungs and the chain length 200 sites. Time steps in these calculations were $\Delta t = 0.1\hbar/J_\perp$ for the ladder and $\Delta t = 0.01\hbar/J_\perp$ for the t-J chain, while bond dimensions were $\chi = 200$ for the ladder and $\chi = 4000$ for the chain. The final times reached were $t_{\max} \simeq 250\hbar/J_\perp$ for the ladder and $80\hbar/J_\perp$ for the chain. The finite-T MPS results shown in the same figures were obtained using the parameters reported above, namely time steps $\Delta t = 0.0625\hbar/J_\perp$ and $\Delta\tau = 0.02\hbar/J_\perp$ for ladders of $L = 45$ rungs, with a bond dimension of $\chi = 620$, and $\Delta t = 0.02\hbar/J_\perp$ and $\Delta\tau = 0.2\hbar/J_\perp$ for chains of length $L = 40$ sites, with a bond dimension of $\chi = 4000$. The final times in all calculations were extended by linear prediction, which reproduced comfortably all the data shown in Figs. 3, 4, 5a-b and 5f-g of the main text, and was particularly valuable in modelling the $T = 150$ mK chain data shown in Figs. 5c-e and 5h-j. MPS spectral data were convolved with the structure factor calculated using Supplementary Eq. (1) and were binned in momentum and energy in the same way as the experimental data (i.e. for time-of-flight data neither a resolution function nor a Gaussian filter is required to model the measured spectrum).

C. Previous studies of t-J chain spectra

For additional context, we comment on previous calculations of the spectral functions of t-J chains, or of near-equivalent Hubbard models. As already noted, the minimal quantum spin ladder simulates a t-J chain with just one parameter ratio, $2t = J$, because both are governed by the leg coupling, $J_\parallel$. This supersymmetric ratio lies rather far from the oft-investigated situation $t \approx 3J$ relevant to cuprate materials, and indeed to most insulating spin chains;[29] to our knowledge, it has also not been considered in ultracold atomic chains.[30] In the regime $t > J$, it is found[21,22] that the continuum-edge excitation (controlled by J) becomes very flat, and the continuum (controlled by t) very wide, for q values in the inner half of the Brillouin zone ($0.00 \leq |q_h| \leq 0.25$), still with no breaking of the continuum into discrete states. In this parameter regime one may apply the terminology of spin-charge separation,[21,22] equating the cosinusiodal features of the continuum directly with the holon response and the flat continuum-edge features with the spinon. By contrast, our results show that the spin and charge degrees of freedom remain strongly entangled throughout the spectrum at the supersymmetric parameter ratio.

Finally, we comment that previous work on the finite-temperature spectral function of the t-J model was also performed for the conventional parameter regime $t/J > 1$.[31] In this situation one observes the physics of the spin-incoherent Luttinger liquid,[32,33] where the spin and charge degrees of freedom are widely separated by energy scale and thus decohere at quite different temperatures.

However, in the supersymmetric t-J model, the characteristic energy scales in both sectors become identical and instead we recover the much more intriguing physics of the pole that is preserved at all temperatures (meaning all temperatures below the effective holon charge gap, set in the ladder-derived t-J model by $J_\perp$).

---

[1] Patyal, B. R., Scott, B. L. & Willett, R. D. Crystal-structure, magnetic-susceptibility, and EPR studies of bis(piperidinium)tetrabromocuprate(II): A novel monomer system showing spin diffusion. *Phys. Rev. B* **41**, 1657–1663 (1990).

[2] Ward, S. *et al.* Spin ladders and quantum simulators for Tomonaga-Luttinger liquids. *J. Phys.: Condens. Matter* **25**, 014004 (2013).

[3] Ward, S. *et al.* Bound States and Field-Polarized Haldane Modes in a Quantum Spin Ladder. *Phys. Rev. Lett.* **118**, 177202 (2017).

[4] Bewley, R. I., Taylor, J. W. & Bennington, S. M. LET, a cold neutron multi-disk chopper spectrometer at ISIS. *Nucl. Instrum. Methods Phys. Res., Sect. A* **637**, 128–134 (2011).

[5] Arnold, O. *et al.* Mantid – data analysis and visualization package for neutron scattering and μSR experiments. *Nucl. Instrum. Methods Phys. Res., Sect. A* **764**, 156–166 (2014).

[6] Tong, D., Lectures on Supersymmetric Quantum Mechanics, https://www.damtp.cam.ac.uk/user/tong/susyqm.html.

[7] Cai, M.-L. *et al.* Observation of supersymmetry and its spontaneous breaking in a trapped ion quantum simulator. *Nature Commun.* **13**, 3412 (2022).

[8] Bares, P. A. & Blatter, G. Supersymmetric t-J model in one dimension: Separation of spin and charge. *Phys. Rev. Lett.* **64**, 2567 (1990).

[9] Bares, P.-A., Blatter, G. & Ogata, M. Exact solution of the t-J model in one dimension at $2t = \pm J$: Ground state and excitation spectrum. *Phys. Rev. B* **44**, 130 (1991).

[10] Essler, F. H. L. & Korepin, V. E. Higher conservation laws and algebraic Bethe Ansätze for the supersymmetric t-J model. *Phys. Rev. B* **46**, 9147 (1992).

[11] Bouillot, P. *et al.* Statics and dynamics of weakly coupled antiferromagnetic spin-$\frac{1}{2}$ ladders in a magnetic field. *Phys. Rev. B* **83**, 054407 (2011).

[12] Batchelor, M. T., Guan, X.-W., Oelkers, N. & Tsuboi, Z. Integrable models and quantum spin ladders: comparison between theory and experiment for the strong coupling ladder compounds. *Adv. Phys.* **56**, 465 (2007).

[13] des Cloizeaux, J. & Pearson, J. J. Spin-Wave Spectrum of the Antiferromagnetic Linear Chain. *Phys. Rev.* **128**, 2131–2135 (1962).

[14] Thielemann, B. *et al.* Direct Observation of Magnon Fractionalization in the Quantum Spin Ladder. *Phys. Rev. Lett.* **102**, 107204 (2009).

[15] Lake, B. *et al.* Multispinon Continua at Zero and Finite Temperature in a Near-Ideal Heisenberg Chain. *Phys. Rev. Lett.* **111**, 137205 (2013).

[16] Klanjšek, M. *et al.* Controlling Luttinger Liquid Physics in Spin Ladders under a Magnetic Field. *Phys. Rev. Lett.* **101**, 137207 (2008).

[17] Kestin, N. & Giamarchi, T. Low-dimensional correlations under thermal fluctuations. *Phys. Rev. B* **99**, 195121 (2019).

[18] Ewings, R. A. *et al.* Horace: Software for the analysis of data from single crystal spectroscopy experiments at time-of-flight neutron instruments. *Nucl. Instrum. Methods Phys. Res., Sect. A* **834**, 132–142 (2016).

[19] Schollwöck, U. The density-matrix renormalization group in the age of matrix product states. *Ann. Phys.* **326**, 96–192 (2011).

[20] Kim, C. *et al.* Observation of Spin-Charge Separation in One-Dimensional $SrCuO_2$. *Phys. Rev. Lett.* **77**, 4054–4057 (1996).

[21] Kim, C. *et al.* Separation of spin and charge excitations in one-dimensional $SrCuO_2$. *Phys. Rev. B* **56**, 15589–15595 (1997).

[22] Kim, B. J. *et al.* Distinct spinon and holon dispersions in photoemission spectral functions from one-dimensional $SrCuO_2$. *Nature Phys.* **2**, 397–401 (2006).

[23] Nayak, M., Blosser, D., Zheludev, A. & Mila, F. Magnetic-Field-Induced Bound States in Spin-$\frac{1}{2}$ Ladders. *Phys. Rev. Lett.* **124**, 087203 (2020).

[24] Sorella, S. & Parola, A. Theory of hole propagation in one-dimensional insulators and superconductors. *Phys. Rev. B* **57**, 6444–6473 (1998).

[25] Saiga, Y. & Kuramoto, Y. Dynamical Properties of the One-Dimensional Supersymmetric t-J Model: A View from Elementary Excitations. *J. Phys. Soc. Jpn.* **68**, 3631–3642 (1999).

[26] White, S. R. & Affleck, I. Spectral function for the $S = 1$ Heisenberg antiferromagetic chain. *Phys. Rev. B* **77**, 134437 (2008).

[27] Pereira, R. G., White, S. R. & Affleck, I. Spectral function of spinless fermions on a one-dimensional lattice. *Phys. Rev. B* **79**, 165113 (2009).

[28] Barthel, T., Schollwöck, U. & White, S. R. Spectral functions in one-dimensional quantum systems at finite temperature using the density matrix renormalization group. *Phys. Rev. B* **79**, 245101 (2009).

[29] Matsueda, H., Bulut, N., Tohyama, T. & Maekawa, S. Temperature dependence of spinon and holon excitations in one-dimensional Mott insulators. *Phys. Rev. B* **72**, 075136 (2005).

[30] Kollath, C., Schollwöck, U. & Zwerger, W. Spin-Charge Separation in Cold Fermi Gases: A Real Time Analysis. *Phys. Rev. Lett* **95**, 176401 (2005).

[31] Feiguin, A. E. & Fiete, G. Spectral properties of a spin-incoherent Luttinger liquid. *Phys. Rev. B* **81**, 075108 (2010).

[32] Cheianov, V. V. & Zvonarev, M. B. Nonunitary Spin-Charge Separation in a One-Dimensional Fermion Gas. *Phys. Rev. Lett.* **92**, 176401 (2004).

[33] Fiete, G. The spin-incoherent Luttinger liquid. *Rev. Mod. Phys.* **79**, 801 (2007).
